# Enhancement of Magnetic Hyperthermia by Mixing Synthetic Inorganic and Biomimetic Magnetic Nanoparticles

**DOI:** 10.3390/pharmaceutics11060273

**Published:** 2019-06-11

**Authors:** Guillermo R. Iglesias, Ylenia Jabalera, Ana Peigneux, Blanca Luna Checa Fernández, Ángel V. Delgado, Concepcion Jimenez-Lopez

**Affiliations:** 1 Department of Applied Physics, Faculty of Sciences, University of Granada, 18071 Granada, Spain; lunachecaf@gmail.com (B.L.C.F.); adelgado@ugr.es (Á.V.D.); 2 Department of Microbiology, Faculty of Sciences, University of Granada, 18071 Granada, Spain; yjabalera@ugr.com (Y.J.); apn@ugr.es (A.P.)

**Keywords:** biomimetic magnetite, drug delivery, magnetic hyperthermia, magnetite, MamC, nanoparticles stability

## Abstract

In this work we report on the synthesis and characterization of magnetic nanoparticles of two distinct origins, one inorganic (MNPs) and the other biomimetic (BMNPs), the latter based on a process of bacterial synthesis. Each of these two kinds of particles has its own advantages when used separately with biomedical purposes. Thus, BMNPs present an isoelectric point below neutrality (around pH 4.4), while MNPs show a zero-zeta potential at pH 7, and appear to be excellent agents for magnetic hyperthermia. This means that the biomimetic particles are better suited to be loaded with drug molecules positively charged at neutral pH (notably, doxorubicin, for instance) and releasing it at the acidic tumor environment. In turn, MNPs may provide their transport capabilities under a magnetic field. In this study it is proposed to use a mixture of both kinds of particles at two different concentrations, trying to get the best from each of them. We study which mixture performs better from different points of view, like stability and magnetic hyperthermia response, while keeping suitable drug transport capabilities. This composite system is proposed as a close to ideal drug vehicle with added enhanced hyperthermia response.

## 1. Introduction

In spite of the certainly wide variety of magnetic nanoparticles (MNPs) with different geometries, compositions and functionalizations [1,2,3,4,5,6,7,8] that have become available in recent years, and of the number of applications that have been devised for them [1,9,10,11,12], when the goal is to provide a nanocarrier suitable for targeted chemotherapy, there is still room for progress. On one hand, the methods for obtaining MNPs need to be more cost- and time-effective, eco-friendly, and scalable. On the other, the nanoparticles themselves should be improved in terms of maximizing the magnetic moment per particle and providing novel surface properties while exploring their potential as hyperthermia agents that would allow them to combine therapies in the near future.

In this context, cancer is one of the fields of application where magnetic nanoparticles certainly appear as most promising [12,13,14,15,16,17,18]. Aside of drug delivery, whereby functionalized magnetic nanoparticles are loaded with the chosen drug and some targeting molecule, such an antibody, driven to the site of action, maintained there by continuous magnetic fields and eventually set for delivery by some external action, magnetic hyperthermia appears as a realistic application of MNPs [19,20,21,22,23,24,25,26,27]. For that purpose, MNPs are dispersed at a suitable concentration in an aqueous solution and located in the target place for action. There, they are subjected to an alternating magnetic field (with induction of tens mT and frequency of several hundred kHz). Subsequently, the magnetic nanoparticles increase the temperature of the microenvironment in which they are immersed inducing apoptosis of the tumor cells, usually more sensitive to temperature increase compared to healthy cells [28,29,30].

MNPs are generally produced either by the co-precipitation of iron salts in basic aqueous media possibly stabilized by biocompatible surfactants/polymers or by the thermal decomposition of organometallic precursors in high-boiling nonpolar organic solvents at elevated temperatures (200–360 °C), allowing a great control of the size of the MNPs, their monodispersity and uniformity [31]. However, these methods have some drawbacks mainly derived from the high temperatures used, organic solvents or poor solubility of the nanoparticles in water.

Many of these drawbacks are overcome in biomimetic MNPs (BMNPs). These are produced by the mediation of magnetosome membrane-associated proteins (MAPs) from magnetotactic bacteria, and *in vitro* experiments have been demonstrated to control the size (and thus the magnetic moment per particle), shape, and surface properties of the nanoparticles [32,33]. BMNPs production can be scaled up *in vitro* in eco-friendly, cost-effective magnetite precipitation experiments run at room temperature and 1 atm total pressure by the simple addition of the recombinant protein. Promising BMNPs have been obtained by the mediation of MamC protein from *Magnetococcus marinus* MC-1, since these BMNPs are (i) superparamagnetic at room and body temperature while they present a saturation magnetization of 61 emu/g (at 500 Oe and 25 °C); (ii) are larger than most commercial MNPs and/or other biomimetic magnetites, although still single magnetic domain, showing higher blocking temperature and slower magnetization increase, and thus, larger magnetic moment per particle; (iii) contain up to 4.5 wt% of MamC, which provides functional groups allowing for functionalization; and (iv) adopt the isoelectric point of MamC (pH_iep_ 4.4), and are strongly negatively charged at physiological pH (pH 7.4); this property allows the coupling and release of molecules to be pH-dependent. Thus, at physiological pHs, they bind to positively charged molecules (such as doxorubicin, DOXO hereafter) through electrostatic interactions, which are weaker at acidic pHs (such as those found in tumor microenvironments), allowing the release of the adsorbed molecules, and (v); they are fully cytocompatible and hemocompatible, but when they are coupled with DOXO they display dose-dependent cytotoxicity [33].

Recall that single-domain magnetic nanoparticles are characterized by a spin configuration such that in the absence of an external magnetic field, all spins are oriented parallel to each other and parallel or antiparallel to a crystallographic direction, called the easy (or anisotropy) axis [34,35,36], so that each particle is characterized by a large magnetic moment. Let us imagine for the moment that the particles are immobile, fixed in a non-magnetic matrix, with their easy axes oriented randomly. At very low temperature (or at very high frequencies of the external magnetic field, if this is non-stationary), the transition between the two orientations along the easy axes can only be achieved by the application of sufficiently large external magnetic field, so that the magnetization-H curve will take the form of a square hysteresis cycle, according to the Stoner-Wohlfarth model [35,37]. As temperature rises above 0 K, the transition between the two orientations can be thermally activated, and the coercivity tends to be reduced, eventually making hysteresis negligible. The same effect will be observed when the field frequency or the particle size is very low, since the coercive field *H*_C_ is related to the anisotropy field *H_k_* (in turn related to *K*_eff_, the effective magnetic anisotropy constant of the particles, and their saturation magnetization *M*_S_ as *H_k_* = 2 *K*_eff_ /μ_0_*M*_S_) by [35]:

(1) $$H_{C}=0.48H_{K}(1-\kappa^{0.8})$$

with

(2) $$\kappa =\frac{k_{B}T}{K_{\mathrm{eff}}V}\ln\left(\frac{k_{B}T}{4\mu_{0}H_{0}M_{S}fV\tau_{0}}\right)$$

where *k_B_T* is the thermal energy, *V* is the particle volume (or, in the case of coated particles, the volume of the magnetizable core), *μ*_0_ is the vacuum magnetic permeability, *H*_0_ is the field amplitude, *f* is the field frequency, and the characteristic time *τ*_0_ depends on such quantities as temperature, saturation magnetization or anisotropy constant. It will be assumed to be a constant in the range of 10^−10^–10^−9^ s. If it is admitted that it suffices with *H*_C_ being 0.01–0.1*H*_k_ to have reversible cycles and absence of hysteresis, then the combination of *H*_0_, *f*, *V*, and *M*_S_ must be such that *κ* = 0.97–0.75.

An approximate approach to the general solution of the problem, not using models based on the Stoner-Wohlfarth one is the so-called linear-response theory, where, keeping the assumption of immobile particles, it is found that the hysteresis cycle has its origin in the Néel-Brown relaxation, characterized by a relaxation time *τ*_N_, which is a measure of the time taken by the system to return to equilibrium after application of a step magnetic field, or half the time needed for spontaneous inversion of magnetic moment orientation [38,39,40]. It is given by:

(3) $$\tau_{N}=\tau_{0}\exp\left(\frac{K_{\mathrm{eff}}V}{k_{B}T}\right)$$

This brings about a delay between magnetization and field, or an imaginary component of the magnetic susceptibility, and manifests again in a finite area hysteresis cycle, as long as the frequency of the field remains in the vicinity of 1/*τ*_N_. This approach is only strictly valid for low applied magnetic field strength or highly anisotropic particles.

For hyperthermia applications, the particles are typically dispersed in an aqueous solution, and hence they can rotate under field inversions so that viscous friction is an additional source of phase delay between magnetization and external field, and hence, an additional relaxation contribution to hysteresis. It is called Brownian relaxation, and it is characterized by a time *τ*_B_:

(4) $$\tau_{B}=\frac{3\eta V_{H}}{k_{B}T}$$

where *η* is the viscosity of the medium, and *V*_H_ its hydrodynamic volume (including, if any, that of the coating layer) [19,41,42]. Taking the value of *K*_eff_ equal to 25 kJ/m^3^ for magnetite, the Brownian relaxation times for biomimetic (~35 nm in diameter, reported below) and purely inorganic particles (~28 nm) are, respectively, 14.4 μs, and 7.4 μs, and orders of magnitude higher in the case of magnetization reversal. This means that for the particle sizes and frequencies involved, the magnetic moment is frozen in the particle and the only heating source is friction, according to the linear response model. Considerations on the validity of the relaxation approach just described can be found in References [43,44,45,46]. In the context of the linear response theory, if the alternating magnetic field has a frequency in the vicinity of the reciprocal of the mentioned times (*f* in the order of 70 kHz and 135 kHz, respectively), the imaginary component, *χ*’’, of the complex magnetic susceptibility is maximum, and this is important, as the dissipated power d*W*/d*t* is proportional to this quantity [28]:

(5) $$\frac{dW}{dt}=\mu_{0}\pi \chi^{\prime \prime }H_{0}^{2}$$

Another important aspect, not always considered, regards the stability of the particles in the suspension, compromised not only by the colloidal interactions but also by the magnetic dipolar ones. Hence the need for properly controlling the stability, as the hyperthermia response degrades for aggregated systems [22,47] or when the monodomain range is surpassed [36,48].

Experimentally, the quantity of interest is the so-called Specific Absorption Rate (SAR), or heat released per unit mass of magnetic material (*m*) and per second:

(6) $$SAR=\left(\frac{CV_{s}}{m}\right)\frac{dT}{dt}$$

*C* being the volume heat capacity of the suspension, *V_s_* its volume and d*T*/d*t* the rate of temperature increase [28,39]. Typical *SAR* values are in the order of tens to hundreds of W/g. In order to perform a comparison between different materials without the interference of the details of the specific device used (very often lab-made), a quantity is defined as a measure of the magnetothermal performance of a given suspension, namely, the Intrinsic Loss Power (ILP), given as:

(7) $$ILP=\frac{SAR}{fH_{0}^{2}}$$

with typical values in the order of 10^−9^ Hm^2^kg^−1^.

In this work, we explore the possibility of maximizing the hyperthermia effect (the rate of temperature rise, in fact) by combining the two types of magnetic particles (BMNPs and MNPs), differing in size and other properties. The goal of the present study is to provide a composition that could be used in the future as a platform for combining drug delivery and hyperthermia. The former particles, BMNPs, mediated by MamC have been chosen because of their demonstrated ability to function as nano-transporters of drugs, being the nanoassembly stable at physiological pH while it destabilizes releasing the drug under acidic pH values, which naturally occur in the tumor environment. The second ones, MNPs, have been chosen because of their potential as hyperthermia agents [33]. By mixing the two systems, we expect to deliver a system that, in the future may prove useful to combine both treatments, i.e targeted chemotherapy plus targeted hyperthermia by using the same platform. To the best of our knowledge, the hyperthermia of mixed systems has never been investigated, but advantages are foreseen regarding the possibility of optimizing the response.

## 2. Experimental

### 2.1. BMNPs and MNPs Production

Details of the production process are given in Reference [32], and just a short account will be provided here. The MamC gene (ABK44766.1, NCBI Database) was first amplified by the polymerase chain reaction and cloned into pTrcHis-TOPO vector, and expressed in *Escherichia coli* TOP10 competent cells, both from Life Technologies, Invitrogen, Grand Island, NY, USA. The cells were grown at 37 °C in Luria-Bertani (LB) broth supplemented with 100 mg/mL of ampiciline. After 5-h contact with isopropyl-β-d-thiogalactopyranoside (IPTG, Fisher BioReagents, Pittsburgh, PA, USA) the expression of the recombinant MamC was induced. The cell pellet was resuspended in guanidinium lysis buffer overnight. The lysate was centrifuged, and the supernatant loaded onto a HiTrap chelating HP column (GE Healthcare, Chicago, IL, USA) equilibrated with Denaturing Binding Buffer (A). The protein eluate was obtained with Denaturing Elution Buffer (B), and the final step was isolating the MamC protein by successive dialyzations with buffers A and B. The isolated protein was stored at 4 °C until used in the biomineralization process (all buffers were purchased from Sigma Aldrich, St. Louis, MO, USA).

The following step was the production of the MNPs and BMNPs (all reagents needed in this process were purchased from Sigma-Aldrich, Madrid, Spain). Milli-Q water (Millipore, Barcelona, Spain) was first deoxygenated by boiling it for 1 h and then cooling in an ice bath while bubbling nitrogen. The water was immediately stored inside an anaerobic chamber (Coy Laboratory Products, Grass Lake, MI, USA) with a 4% H_2_ in N_2_ atmosphere. The following solutions were prepared inside the chamber: NaHCO_3_/Na_2_CO_3_ (0.15 M/0.15 M), FeCl_3_ (1 M), Fe(ClO_4_)_2_ (0.5 M), and NaOH (5 M). The precipitation of inorganic magnetite (MNPs) was carried out as described in Reference [49], and consisted in mixing the prepared solutions to the final concentrations of 3.5 mM/3.5 mM, 5.56 mM, and 2.78 mM, respectively, at 25 °C and 1 atm. NaOH was added to reach a pH of 9. The biomimetic nanoparticles were obtained by adding the purified MamC protein (at a final concentration of 10 µg/mL) to the solution used for MNPs. In both cases, the solids were allowed to grow for 30 days, and magnetically decanted and washed three times with deoxygenated water. The magnetic particles were kept in water inside the Coy chamber until further use.

### 2.2. Nanoparticle Characterization

The morphology and particle size of the synthesized nanocrystals were analyzed by Transmission Electron Microscopy (TEM Philips Model CM20, Eindhoven, The Netherlands) equipped with an energy dispersive X-ray spectrometer (EDAX). The size of the particles was measured by using ImageJ 1.47 software, and size distribution curves and ANOVA statistical analyses were determined from measurements performed on 1000 particles. Averages were considered significantly different if *p* < 0.05. Powder x-ray diffraction (XRD) analysis was carried out on lyophilized samples with an Xpert Pro X-ray diffractometer (PANalytical; Almelo, The Netherlands) using Cu Kα radiation, with the scan range set from 20–60° in 2*θ* (0.01°/step; 3 s per step). Electrophoretic mobility measurements were carried out in a Zetameter Nano-ZS (Malvern Instruments, Malvern, UK) at 25 °C, in suspensions with 0.01% *w*/*v* solids concentration and constant ionic strength of 5 mM KNO_3_. For each suspension, 5 measurement runs were taken. Magnetization cycles and zero-field cooled field-cooled (ZFC-FC) curves were obtained in an MPMS-XL SQUID magnetometer (Quantum Design, San Diego, CA, USA). The stability of the samples was evaluated optically by measuring the time evolution of the phase separation line between particles and medium: The samples were photographed at certain intervals. Afterward, the height and volume of each phase were determined through image processing and analyzed.

Magnetic hyperthermia experiments were carried out using an AC current generator with a double four-turn coil made of water-cooled copper tube, 4 mm in inner diameter, with 800 mL/min flow rate, comparable to other experimental hyperthermia devices [50]. Three frequencies, namely, 197 kHz, 236 kHz, and 280 kHz were selected, with a fixed magnetic field intensity of 18 kA/m, measured at the center of the coil with a NanoScience Laboratories Ltd. Probe (Staffordshire, UK), with 10 μT resolution. These were the combinations accessible with our measurement system, but are close to those used by other authors [45,46,51]. The samples to be evaluated were placed in plastic Eppendorf tubes (1.5 mL sample volume). Four kinds of dispersed systems were evaluated, namely those based on pure MNPs or BMNPs, and mixtures containing 25% BMNPs + 75% MNPs (here referred to as 25 B + 75 M), and 60% BMNPs + 40% MNPs (60 B + 40 M). This selection was based on using a combination with a predominance of inorganic MNPs and another one with a higher fraction of biomimetic particles in order to estimate the relative contribution of each type of particles.

The *SAR* and *ILP* of the dispersions were obtained by measuring the rate of temperature increase as a function of time [19,52], with an optical fiber thermometer (Optocon AG, Dresden, Germany), and using Equations (6) and (7). Considering the rather low concentration of MNPs, the corrections proposed by Gas and Miaskowski [51] in the calculation of the heat capacity of the suspension did not appear necessary. All samples for hyperthermia were prepared with a solids concentration of 25 mg/mL. Note that this concentration is higher than usual in hyperthermia applications (more in the range of 1–10 mg/mL), but it was chosen with the aim of magnifying differences. At such concentrations, magnetic or colloidal interactions might likely affect hyperthermia, as increased stability seems to favor the temperature elevation, if the frequency of the magnetic field is selected in accordance with the size of individual, non-aggregated particles. In fact, in a previous work [22], we found that the hyperthermia response was improved if the suspensions were more stable, although the *SAR* of 20 nm magnetite suspensions was constant up to 2% (*v*/*v*) (or about 100 mg/mL) concentration.

## 3. Results and Discussion

### 3.1. Particle Characterization

**Figure 1:**
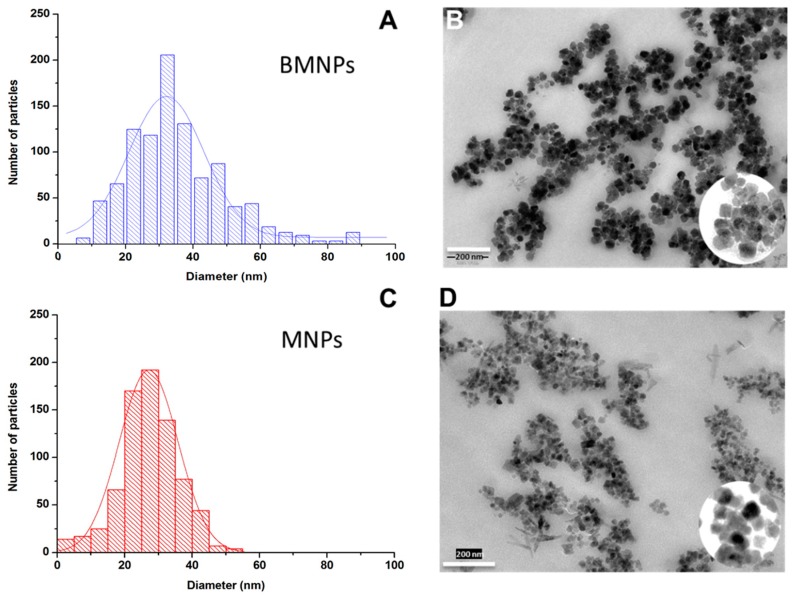
Diameter histograms (**A**,**C**) and TEM pictures (**B**,**D**) of biomimetic (BMNPs) and purely inorganic (MNPs) magnetic nanoparticles. The scale bar in B, D is 200 nm.

Figure 1B,D show representative TEM pictures of the two kinds of particles. Size histograms are represented in Figure 1A,C, respectively. The mean (± S.D.) diameters obtained from the histograms were 35 ± 11 nm for BMNPs and 18 ± 6 nm for the purely inorganic nanoparticles. Insets in the pictures also show that the shape of both kinds of particles is rather polyhedral, with better homogeneity in the biomimetic case.

**Figure 2:**
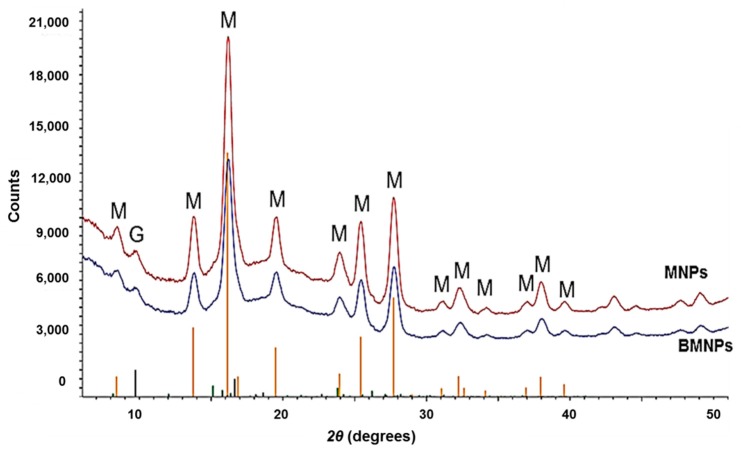
The XRD diffraction patterns of purely inorganic (MNPs) and biomimetic magnetic nanoparticles (BMNPs). The positions and intensities of crystallographically-pure magnetite are labeled as M, and those of goethite as G.

Figure 2 shows the XRD patterns of both samples. Note the good crystallinity in the two cases, and the excellent coincidence with the magnetite reference pattern (JCPDS card No 19-0629), being the main reflection for magnetite the 311 (d-spacing = 2.530 Å, for Cu Kα radiation, 2θ is 35.44 degrees). The goethite diffraction lines are also superimposed in the figure, and only a low-angle peak seems to correspond to this oxide. Using Scherrer’s formula [53], the crystallite size was obtained for both samples from the half-intensity width of all the lines in the pattern, using specialized Rietveld software (TOPAS 5.0, Bruker, Hamburg, Germany). Calculated crystallite sizes for MNPs vary between 13.0 and 18.3 nm, while those for BMNPs vary between 13.1 and 22.5 nm. Thus the calculated size from XRD data for MNPs matches that measured from TEM images. However, this is not the case for BMNPs for which we get smaller values than those measured with TEM. Recall that the crystallite size calculated from XRD data is a measure of the size of coherent diffraction domains, which can be smaller than the particle size if small discontinuities will make the domain lose such a coherency. It is true that BMNPs could be polycrystals, but this is not what was found by High-Resolution TEM observations of BMNPs in Reference [54]. No discontinuities in lattice fringes were observed, and therefore, the polycrystallinity of BMNPs seems to be ruled out, at least for the majority of the BMNPs analyzed. However, Garcia-Rubia et al. [33] suggested the incorporation of MamC in the outer layers of the BMNPs crystals, that prevented the removal of the protein, and measured, in fact, that 5% of the total mass of the BMNPs is MamC. The presence of the protein would, most probably, induce some defects in the crystal structure, resulting in the loss of coherency in the diffraction. This is why, in BMNPs, the crystal sizes calculated from XRD are lower than those measured in TEM images.

**Figure 3:**
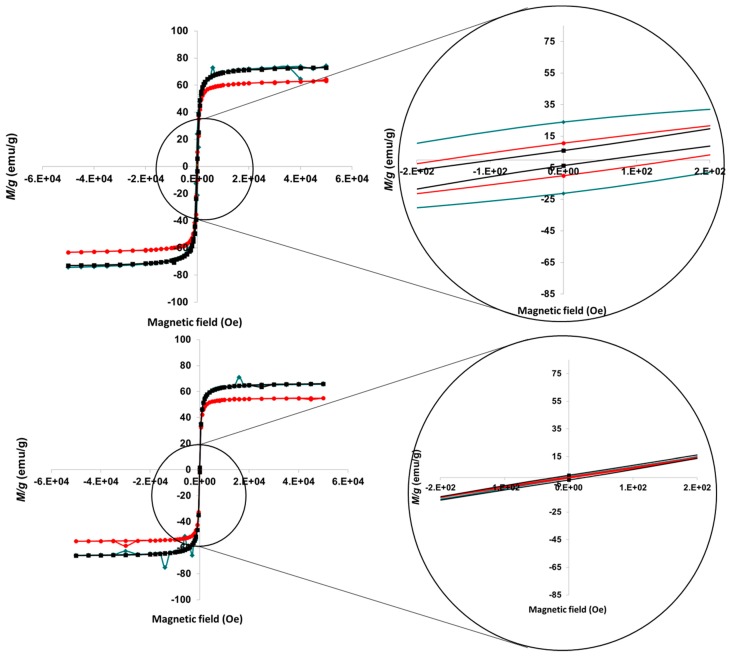
Magnetization cycles of MNPs (■), BMNPs (●), and 25 B + 75 M (♦) at 5 K (**top**), and 300 K (**bottom**). Magnifications of the low-field region are also plotted.

The magnetization of the two kinds of particles and an example of one of the mixtures (25 B + 75 M, in fact the most stable mixture, as will be discussed below) is plotted in Figure 3 for two temperatures, 5 K and 300 K. The detail in Figure 3 shows that some really low remnant magnetization (about 20 emu/g at most), and coercivity can be measured at 5 K, with the interesting feature that this is maximum for the mixed system. This can be ascribed to some degree of aggregation between the two kinds of particles, as at the pH of the aqueous suspensions in which the mixtures were prepared they are oppositely charged (detailed below), and electrostatic attraction cannot be ruled out. For room temperature measurements, the magnetization shows no hysteresis, and the particles behave as paramagnetic. This is characteristic of superparamagnetism, as mentioned. At room temperature, the saturation (mass) magnetization reaches 66 emu/g in the case of MNPs and 25 B + 75 M, and 55 emu/g in the case of BMNPs. Considering the dilution effect of the coating caused by the incorporation of MamC [33], the corrected value of saturation magnetization is 61 emu/g for BMNPs, and 70 emu/g for 25 B + 75 M.

**Figure 4:**
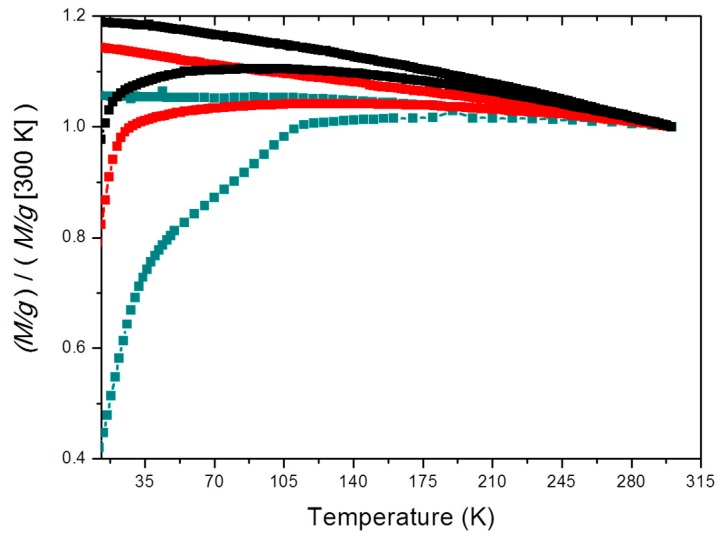
Zero field cooling-field cooling (ZFC-FC) curves at 40 kA/m of MNPs (■), BMNPs (●) and 25 B + 75 M (♦).

Zero-field cooling-field cooling (ZFC-FC) curves at 500 Oe (39.8 kA/m) (see Figure 4) show that the blocking temperature (maximum in the ZFC cooling curve, the lower branch in each case, corresponding to the rounded appearance of the curve [55]) is 103 K for MNPs, 145 K for BMNPs and 180 K for 25 B + 75 M, and that at temperatures higher than blocking temperature, (including 300 K and above in all cases), these particles will behave as non-magnetic in the absence of an external magnetic field, confirming the superparamagnetic nature of the two kinds of particles. This prevents magnetic aggregation, a very favorable feature of the systems investigated. 

However, once an external magnetic field is applied, the nanoparticles will respond efficiently. From these results, it can be inferred that either the bare MNPs and BMNPs, or their mixtures, appear as ideal candidates for the purpose of hyperthermia, drug delivery or combinations thereof.

### 3.2. Zeta Potential

**Figure 5:**
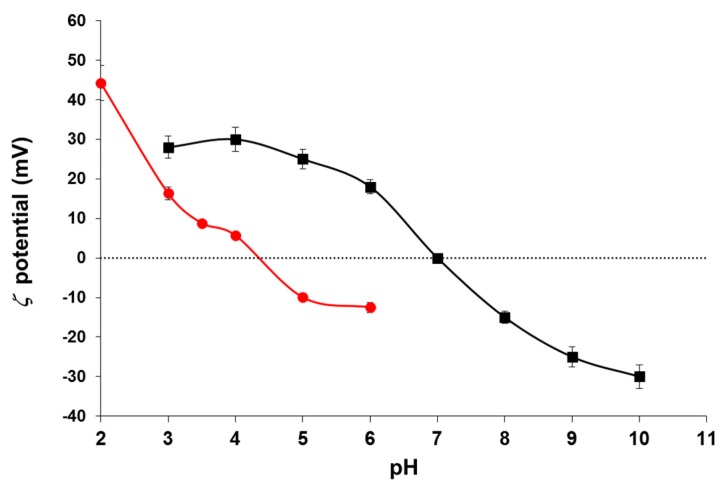
Zeta potential of purely inorganic MNPs (■) and biomimetic magnetic nanoparticles BMNPs (●).

For the purpose of drug loading and delivery, the ability of the nanoparticles to be coupled to molecules forming stable nanoassemblies at physiological pH and to release the drug at the precise time, as well as the stability, drug-particle or cell membrane-particle interactions, among other properties, are strongly determined by the surface charge, or, alternatively, by the zeta potential *ζ*, of the particles when dispersed. Calculations of the zeta potential were done from the measurements of the electrophoretic mobility of the particles BMNPs and MNPs by means of O’Brien and White’s general theory [56]. As one can see in Figure 5, the isoelectric point (pH_iep_ or pH of zero *ζ*) of MNPs was obtained at pH 7.0, while for BMNPs it was 4.4. This zeta potential plot reveals significant differences between both types of nanoparticles. They are positively charged at low pH values and negatively charged at high pH, but BMNPs change from positive to negative at quite a different pH: This suggests that the MamC protein is affecting the surface of the particles, and it is probably located mainly at the interface. In fact, the investigations by Nudelman et al. [57], and other authors [54,58] on the biomineralization mechanism induced by this protein demonstrate that the particularity of MamC is the template effect for magnetite nucleation and growth that this protein exerts. The two negatively charged amino acids Asp70 and Glu66, located in MamC loop, are separated by about 8 Å, and this distance can match the disposition of iron cations on (100), (110), and (111) faces. Therefore, the crystal could grow in an orderly manner, first from the Fe cations available in solution and then, since the amount of Fe in solution is limited, at the expense of the release of the Fe cations adsorbed in other negatively charged moieties of MamC. Since the process of nucleation is kinetically favored by this template effect, it is precisely those exposed amino acids that act as nucleation sites. Therefore, the restricted number of nucleation sites in the presence of MamC compared to the process of homogeneous nucleation from the bulk solution allows the formation of larger crystals in the former. Moreover, since (100), (110), and (111) faces show up in the final morphology of the BMNPs [57], it can be concluded that, while exerting this template effect for the nucleation, the protein prevents the growth of the crystal on these specific directions.

The shift of the isoelectric point of the BMNPs relative to that of MNPs make the former adequate nanocarriers, a fact that is important for the sought application. Since most common drugs for cancer therapy are positively charged at physiological pH, they could be electrostatically attached to BMNPs at such pH values, resulting in a stable nanoassembly. On the contrary, when they eventually reach the tumor (acidic pH values), the drug releases from the BMNPs, as the charge of the latter at acidic pH values is nearly zero [33]. That makes the BMNPs better drug nanocarriers compared to MNPs, as in the former the release of the drug can be controlled by an external factor, as it is a change in the environmental pH value. This feature is absent in MNPs, as their charge is positive anywhere below pH 7.

### 3.3. Stability

**Figure 6:**
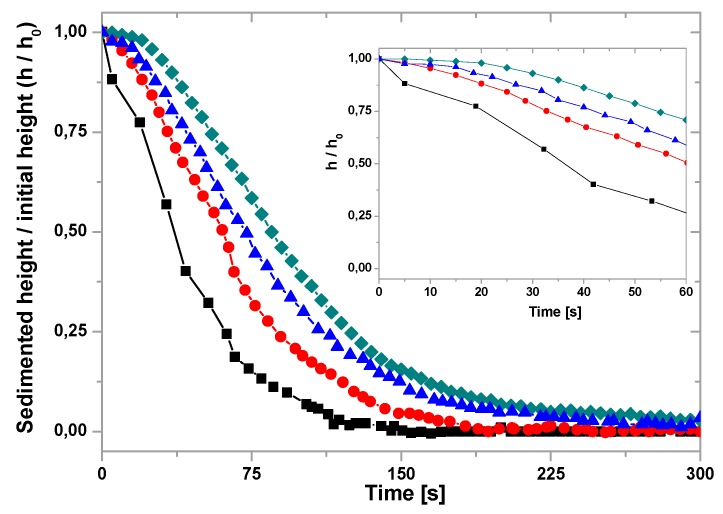
Height of the sedimented volume relative to its initial value as a function of time for MNPs (■), BMNPs (●), 60 B + 40 M (▲), and 25 B + 75 M (♦). Inset: Short-time detail.

Like in many other applications, the use of magnetic nanoparticles in health applications requires to ensure sufficient stability. Previous results from our laboratory demonstrated that, specifically when it comes to hyperthermia applications, the stability, either electrostatically or polymerically achieved, is an essential factor for optimizing the performance of the system [22]. Hence, in this part of the study, we focus on the possibility of improving hyperthermia by favoring stability. It must be recalled that superparamagnetic nanoparticles interact attractively through van der Waals interactions and dipolar magnetic ones (if remanence is not negligible, which is not our case at room temperature, see Figure 3). Furthermore, the repulsive interactions due to the surface charge will be very low at physiological pH values in the case of MNPs, and larger in the case of BMNPs (Figure 5). In addition, steric hindrance could contribute to the stability of the biomimetic nanoparticles through the presence of surface MamC molecules. In order to confirm this possibility, we evaluated the sedimentation behavior vs. time of the samples (pure end members and mixtures of BMNPs and MNPs). The time evolution of the boundary between sedimented volume (height *h*) and clear supernatant, relative to the initial height *h*_0_, will be used as a simple test of stability. Data are plotted in Figure 6. Surprisingly, it can be observed that, although the stability of bare MNPs is lower than that of BMNPs, as expected from the steric and electrostatic repulsions due to the MamC coating, adding BMNPs to the former at a relative concentration of 25/75 (25 B + 75 M) brings about a measurable increase of stability. This may be the result of a compromise between the smaller size of MNPs favoring stability and the addition of the protective coating represented by the BMNPs. As a result, an ideal system with a mixed composition emerges.

### 3.4. Performance in Hyperthermia

**Figure 7:**
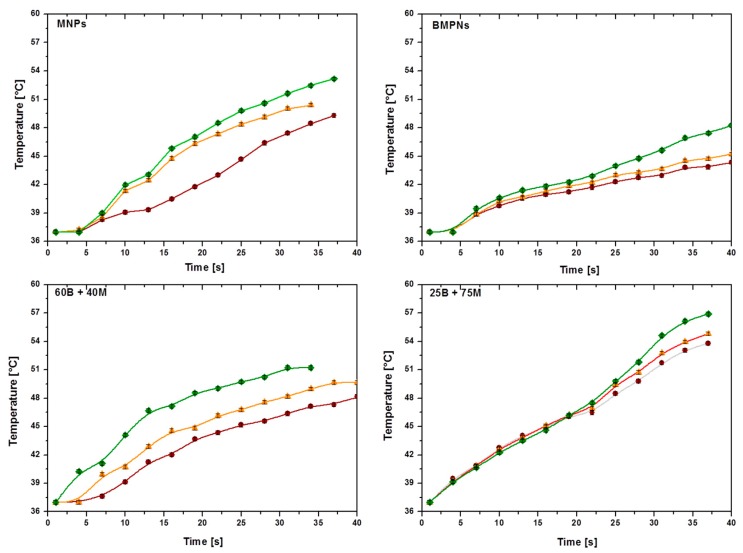
Time evolution of the temperature of the MNP suspensions, for different frequencies: 197 kHz (●), 236 kHz (▲), and 280 kHz (♦). Field strength: *H*_0_ = 18 kA/m. Sample volume 0.5 mL; particle concentration: 25 mg/mL.

As a novel field of application of the two kinds of particles and their mixtures, we consider to what extent they are efficient magnetic hyperthermia agents. Figure 7 shows the time evolution of the temperature of the suspensions of MNPs, BMNPs and the two mixtures 25 B + 75 M and 60 B + 40 M under the influence of an alternating magnetic field at the indicated frequencies and a fixed magnetic field strength of 18 kA/m. All types of nanoparticles are able to raise the temperature, the fastest rise occurring for the highest frequency. This effect is particularly visible at longer times. The rate of temperature increase is maximum (~34 °C/min) in mixture 25 B + 75 M and minimum in BMNPs (~17 °C/min) at the highest frequency. It appears that inorganic MNPs are better hyperthermia agents than BMNPs. This justifies their presence in the mixtures under study. They make it possible to design a composition of nanoparticles that (i) can be guided to the target by the application of an external magnetic field; (ii) behave as suitable drug nanocarriers, stable at physiological pH values and from which the drug release is dependent of an external stimuli, e.g., acidic tumor environment (these are the BMNPs); (iii) produce a fast increase of the temperature of the system upon the application of an external magnetic field (these are the MNPs).

**Figure 8:**
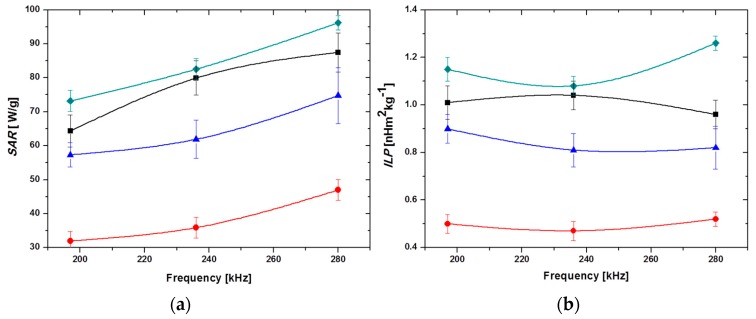
Frequency dependence of (**a**) *SAR* and (**b**) *ILP*, for the investigated systems. MNPs (■), BMNPs (●), 60 B + 40 M (▲), and 25 B + 75 M (♦). Magnetic field strength: 18 kA/m; particle concentration: 25 mg/mL.

In fact, maximum *SAR* (up to 96.2 W/g) and *ILP* (1.26 nHm^2^kg^−1^) values were obtained for the 25 B + 75 M sample, followed by MNPs, 60 B + 40 M and, finally, BMNPs (Figure 8, Table 1). The lower heating induced by BMNPs is probably related to the larger size of the particles and to the presence of MamC attached to their surface, which probably hinders the rotation of the nanoparticles. The increased *SAR* and *ILP* values obtained for the 25 B + 75 M samples is promoted by stability, as single particles with close-to-spherical symmetry rotate under the action of the field less impeded than in the case of aggregated particles. In addition, it is likely that the field frequencies used are closer to those of maximum phase lag between magnetization and field, for the MNPs than for the BMNPs, so the latter would play here the role of favoring stability.

## 4. Conclusions

The present study intended to find an appropriate combination of magnetic nanoparticles that provide suitable targeted hyperthermia, and potentially proper conditions for being loaded with a positively-charged drug at a neutral pH while releasing it at the acid tumor environment. Both inorganic (MNPs) and biomimetic (MamC-mediated, BMNPs) nanoparticles have been proven to be superparamagnetic, having a blocking temperature lower than 300 K, therefore behaving as paramagnetic at this temperature (thus preventing magnetic agglomeration) but exhibiting a relatively high saturation magnetization in the presence of an external magnetic field, being thus able to be magnetically guided to a selected target site. BMNPs have been found to bind different molecules based on electrostatic interactions, forming stable nano-assemblies at physiological pH, based on the change in the isoelectric point (iep) of the nanoparticles induced by MamC (iep = 4.4). In contrast, MNPs are not as good candidates for that purpose, since their pH_iep_ is around 7. The adsorption of a positively charged drug as doxorubicin on BMNPs would be favored at neutral pH, and at the same time its release would also be enhanced as the system acidifies (like in tumor microenvironments) approaching the iep of the BMNPs. However, while potentially good nanotransporters, BMNPs are not as good agents for hyperthermia as MNPs. Therefore, the present study offers a composition of nanoparticles, namely, 25% BMNPs + 75% MNPs that, while having the maximum hyperthermia response, likely related to improved stability of the sample, is also suitable as a drug nanocarrier designed to deliver the drug in response to changes in the environmental pH. Therefore, the combination of inorganic and biomimetic nanoparticles potentially allows combined targeted chemotherapy and targeted hyperthermia.

## Figures and Tables

**Table 1:** Summary of Specific Absorption Rate (SAR), Intrinsic Loss Power (ILP) calculations, and temperature increase after 60 s exposition time, Δ*T*, for the different samples tested [inorganic Magnetic Nanoparticles (MNPs) and MamC-medianted Biomimetic Magnetic Nanoparticles (BMNPs)], and the field frequencies indicated.

System	Frequency *f* [kHz]	*SAR* [W/g]	*ILP* [nHm^2^kg^−1^]	Δ*T* [°C]
MNPs	197	64 ± 5	1.01 ± 0.07	20.2 ± 0.2
	230	80 ± 5	1.04 ± 0.06	25.2 ± 0.2
	280	87 ± 6	0.96 ± 0.06	28.3 ± 0.2
BMNPs	197	32 ± 3	0.50 ± 0.04	11.6 ± 0.2
	230	36 ± 3	0.47 ± 0.04	12.9 ± 0.2
	280	47 ± 3	0.52 ± 0.03	16.7 ± 0.2
25 B + 75 M	197	73 ± 3	1.15 ± 0.05	28.5 ± 0.2
	230	83 ± 3	1.08 ± 0.04	30.5 ± 0.2
	280	96 ± 2	1.26 ± 0.03	34.1 ± 0.2
60 B + 40 M	197	57 ± 4	0.90 ± 0.06	18.1 ± 0.2
	230	62 ± 6	0.81 ± 0.07	21.6 ± 0.2
	280	75 ± 8	0.82 ± 0.09	27.5 ± 0.2
